# Mass spectrometry imaging of amino neurotransmitters: a comparison of derivatization methods and application in mouse brain tissue

**DOI:** 10.1007/s11306-015-0926-0

**Published:** 2016-01-08

**Authors:** Clara Esteve, Else A. Tolner, Reinald Shyti, Arn M. J. M. van den Maagdenberg, Liam A. McDonnell

**Affiliations:** Center for Proteomics and Metabolomics, Leiden University Medical Center, Einthovenweg 20, 2333 ZC Leiden, The Netherlands; Department of Neurology, Leiden University Medical Center, Leiden, The Netherlands; Department of Human Genetics, Leiden University Medical Center, Leiden, The Netherlands; Fondazione Pisana per la Scienza ONLUS, Pisa, Italy

**Keywords:** Mass spectrometry imaging, Neurotransmitters, Chemical derivatization, Amino acids, Mouse brain

## Abstract

The detection of small polar compounds such as amino neurotransmitters by MALDI mass spectrometry imaging has been hindered by low-detection sensitivity and background interferences. Recently, several of on-tissue chemical derivatization strategies have been independently reported that enable their detection. Here, we present a comparison between these methods, and demonstrate the visualization of the distributions of up to 23 amino metabolites in tissue. We applied this methodology to detect alterations of these compounds after inducing an experimental cortical spreading depression in mouse brain, which causes profound transient alterations in key neurotransmitters in one hemisphere and is relevant for migraine and various other neurological disorders.

**Electronic supplementary material:**

The online version of this article (doi:10.1007/s11306-015-0926-0) contains supplementary material, which is available to authorized users.

## Introduction

Neurotransmitters (NTs) play a vital role in nerve cell communication and regulate a variety of biological processes and behaviours (Lowe 2011). Several NTs are small polar compounds containing one amino group, like amino acids and monoamines. Abnormal concentrations of this neurotransmitters and consequent dysfunction of neural systems are linked to various central nervous system disorders such as schizophrenia (Hirvonen and Hietala 2011), Parkinson’s disease (Klein et al. 2010), Alzheimer’s disease (Lanari et al. 2006), migraine (D’Andrea and Leon 2010), and depression (Mitani et al. 2006). Visualization of changes in the concentrations of NTs in situ will be essential in understanding their role in various neurophysiological processes in different regions of the brain, but until recently has not been feasible.

The advent of mass spectrometry imaging (MSI) as a tool to determinate the distribution of small molecules present in tissue samples (Norris and Caprioli 2013; Fujimura and Miura 2014; McDonnell and Heeren 2007), has opened the door to apply the technology also to the detection of amino neurotransmitters. MSI allows spatially-correlated mass spectrometry analysis to simultaneously assess the distributions of panels of biomolecules in tissue sections. Determination of the spatial localization of NTs by MSI would allow us to directly examine their role within pathological processes, thereby contributing to a better understanding of the pathophysiology of the brain (Miura et al. 2010; Sugiura et al. 2012, 2014). However, the analysis of amino metabolites by MSI has proven difficult because of their low-ionization efficiency, spectral interferences from MALDI matrix-background ions or tissue components, ion suppression effects and analyte in-source fragmentation.

Chemical derivatization is a well-established strategy for the detection of amino metabolites using capillary electrophoresis, gas chromatography and high-performance liquid chromatography (Zhang et al. 2014; Denoroy et al. 2008; Yoon 2013). Recently such methods have begun to be applied to MSI (Toue et al. 2014; Manier et al. 2014; Shariatgorji et al. 2014, 2015) but importantly no systematic comparisons have been performed. Through derivatization the amino neurotransmitters are more easily and more uniformly ionized, and their mass increased; the result is increased detection sensitivity for a range of amino neurotransmitters and less background ions interfering with the analysis.

Here we have systematically compared the recently reported on-tissue chemical derivatization protocols in order to improve the detection of amino metabolites and neurotransmitters by MSI. The optimized methods were then used for the visualization of amino neurotransmitter levels and distributions following cortical spreading depression (CSD), the electrophysiological correlate of migraine with aura. The lack of lack of clear histopathological features following CSD prevents the histology-defined microdissection workflows used in typical molecular pathology research. Instead we use MSI to annotate tissues based on their MS profiles and thereby identify which regions have altered biomolecular profiles (even if they were unexpected or are not distinct using established histological and histochemical methods). By applying neurotransmitter MSI here we report, for the first time, the first direct measure of altered amino neurotransmitters following CSD, a process tightly implicated in migraine, stroke and traumatic brain injury (Lauritzen et al. 2011).

## Materials and methods

### Chemicals

2,5-Dihydroxybenozic acid (DHB), α-cyano-4-hydroxycinnamic acid (CHCA), *N*,*N*′-dihydroxysuccinimidyl carbonate (DSC), *N*,*N*-dimethyl-amino-*p*-phenylenediamine, trifluoroacetic acid (TFA), 4-hydroxy-3-methoxycinnamaldehyde (CA), 2,3-diphenyl-pyranylium tetrafluoro-borate (DPP-TFB), poly-l-lysine solution, acetonitrile (ACN) and methanol (MeOH) were obtained from Sigma-Aldrich (St. Louis, MO, USA). The standards of glycine, cysteine, leucine, glutamine, tryptophan, dopamine, and norepinephrine were also purchased from Sigma-Aldrich. Indium tin oxide (ITO)-coated glass slides were purchased from Bruker Daltonics (Bremen, Germany). *p*-*N*,*N*,*N*-trimethylammonioanilyl *N*-hydroxysuccinimidyl carbamate iodide (TAHS) was synthesized as descried previously (Shimbo et al. 2009).

### Animal protocol

Female C57BL/6J mice of 3 months of age were used. Non-treated mouse brain was used for method development. Cortical spreading depression (CSD) was evoked by topical KCl application to the cortex (SHAM experiments used NaCl, which does not induce CSD events) and monitoring was performed as described previously (Shyti et al. 2015). In brief, surgery was performed using 1.5 % isoflurane anesthesia in pressurized air. A cranial window was prepared above the occipital cortex of the right hemisphere (ca. 3.5 mm posterior, 2 mm lateral from bregma). For evoking CSD events a cotton ball soaked in 300 mM KCl was placed for 30 s on the dura overlaying the occipital cortex. This was followed by a wash with 150 mM NaCl. Using this approach, seven CSDs were evoked by repeated application of KCl with an interval of 5 min. The CSDs were monitored by recording cortical DC-potential changes via a glass microelectrode that was placed in the frontal sensorimotor cortex (0.5 mm anterior, 2 mm lateral from bregma; depth 300 μm). In case of a SHAM treatment, a cotton ball was soaked in 1 M NaCl instead of KCl. Directly after the last CSD, or after the last application of 1 M NaCl in case of the SHAM animal, the mouse was sacrificed by decapitation and the brain quickly isolated and stabilized prior to freezing using a tissue heat-stabilizor device (Stabilizor™, Denator AB, Sweden). This strategy minimizes metabolite post-mortem degradation and preserves metabolite localisation (Sugiura et al. 2014). The tissues were stored at −80 °C until use. Brain tissues were sectioned at 12 μm thickness using a cryostat (1720 Digital, Leica, Rijswijk, The Netherlands) at −20 °C and thaw-mounted onto an ITO-coated glass slide previously coated with poly-l-lysine (0.05 % in water). Note for the analysis of the SHAM and CSD animals cortical brain sections were selected from the region between CSD induction (ca. 3.5 mm posterior to Bregma) and electrophysiology monitoring (0.5 mm anterior to Bregma).

### Derivatization method

We first explored the derivatization of amino metabolites in solution with TAHS, CA, and DPP-TFB under specific conditions following the recommended methods for similar derivativization reactions (Manier et al. 2011; Shimbo et al. 2009; Toue et al. 2014; Rebane et al. 2012). Standards of glycine, cysteine, leucine, glutamine, tryptophan, dopamine and norepinephrine were used for testing. For derivatization with TAHS, 40 μL of reagent dissolved in ACN (5 mg/mL) were mixed with 40 μL of standards (100 μg/mL) and 120 μL borate buffer (0.2 M, pH 8.8). The mixture was kept at 55 °C during 1 h. For derivatization with CA, 100 μL of reagent (2 mg/mL in MeOH) were mixed with 100 μL of standards and kept at 37 °C for 1 h. In the case of derivatization with DPP-TFB, 100 μL of reagent (2 mg/mL in MeOH) were mixed with 100 μL of standards dissolved in borate buffer (0.2 M, pH 8.65) and kept at RT for 1 h. Before analysis, samples were cooled to RT. A volume of 0.5 μL of the samples was deposited onto the MALDI sample target and air-dried. Next, 0.5 μL of DHB (30 mg/mL in 70 % MeOH, 0.1 % TFA) was deposited atop of the sample and air dried before immediate MALDI-Fourier transform ion cyclotron resonance (FTICR)-MS analysis.

To perform on-tissue derivatization of amino metabolites and neurotransmitters (NTs) the SunCollect sprayer (SunChrom, Friedrischsdorf, Germany) was used. Fresh solutions of derivatization reagents were prepared on the same day. TAHS (5 mg/mL in 50 % ACN) was applied using three layers at a flow rate of 10 μL/min and the brain sections were incubated overnight at 55 °C. For the on-tissue derivatization with CA (4 mg/mL in 50 % MeOH), three layers were sprayed using a flow rate of 10 μL/min. The brain tissue sections were then incubated at 37 °C overnight. Finally, for the reaction with DPP-TFB (5 mg/mL in 100 % MeOH), 5 layers at a flow rate of 10 μL/min were applied. For complete derivatization, the brain sections were kept overnight at RT.

For matrix deposition, fresh DHB (30 mg/mL in 70 % MeOH and 0.1 % TFA) solution was sprayed using two layers at 5 μL/min followed by six layers at 10 μL/min.

### MALDI-MSI analysis

MALDI-MSI sample preparation optimization experiments were performed using an UtlrafleXtreme MALDI-TOF/TOF (Bruker Daltonis Inc., Billerica, MA, USA). The data were acquired in the ion reflection mode using external calibration. For accurate mass analysis a 9.4 T SolariX MALDI-FTICR (Bruker Daltonics) was used. MS data were acquired in positive mode using lock-mass calibration in the range *m/z* 50–500 by averaging signals from 500 laser shots. MSI was performed with a spatial resolution of 125 μm for sagittal sections and 100 μm for coronal sections. The MALDI-TOF average mass spectra were normalized to their total-ion-count (TIC) while no normalization was required for the MALDI-FTICR spectra. Data acquisition, processing, and data visualization were performed using the software suite FlexControl 3.4, ftmsControl 2.0, FlexImaging 4.1 and DataAnalysis 4.2 from Bruker Daltonics.

## Results and discussion

The on-tissue detection of amino metabolites and NTs by MALDI MSI has been notoriously challenging, mainly due to the low-ionization efficiency and the background spectral interferences from the MALDI matrix. In order to increase analysis sensitivity, a strategy is proposed that is based on on-tissue derivatization followed by high-resolution MS measurement. For that purpose, derivatization reagents were chosen that have been demonstrated to enhance the detection of amino metabolites in MALDI MSI. The selected compounds were commercially available derivatization reagents CA (Manier et al. 2014) and DPP-TFB (Shariatgorji et al. 2014, 2015), and home-synthesized derivatization reagent TAHS (Rebane et al. 2012; Shimbo et al. 2009). Derivatization reactions of amino groups with each derivatization reagent are shown in Supplementary Figure 1.

Prior to on-tissue experiments the derivatization reagents were tested in-solution. A mixture of standards of amino acids and amines, (glycine, cysteine, leucine, glutamine, tryptophan, dopamine and norepinephrine) were allowed to react with the derivatization reagents. The resulting derivatives were analysed using MALDI-FTICR-MS; for all derivatization reagents the derivatization led to the formation of [M]^+^ ions with no observable [M]^2+^ or [M + Na]^+^ ions. Peaks corresponding to each derivative were observed, with mass shifts of +177.1022 for TAHS derivatives, +160.05188 for CA derivatives, and +215.0855 for DPP-TFB derivatives. The differences between the theoretical and observed *m/z* values of each derivatized standard compound were <1 ppm.

### Optimization of on-tissue derivatization conditions for amino metabolites MALDI MSI

In order to maximize detection sensitivity and maintain the spatial distribution of amino metabolites in tissue, we optimized the derivatization reaction conditions. The effects of incubation time, derivatization reagent concentration, solvent composition, and amount of reagent solution applied to the tissue were optimized for each of the three derivatization reagents. Besides this, the absence of matrix and different organic matrices were also tested.

Reaction temperature was chosen according to the parameters suggested in the published methods (Manier et al. 2014; Shariatgorji et al. 2014; Toue et al. 2014). Derivatization reactions were kept overnight at 55 °C for TAHS, which is necessary to suppress an undesired side reaction between TAHS and the phenolic hydroxyl group of Tyr (Toue et al. 2014). Based on the experience of Manier et al. (2014) a temperature of 37 °C was used for CA reaction. The TPP-TFB reaction was performed at RT, as reported by Shariatgorji et al. (2014). To date no comparison of these three derivatization methods has been reported.

The optimal concentration of derivatization reagent was investigated by achieving the highest detection sensitivity but without the negative effect of large interferences from derivatization reagent peaks. TAHS was dissolved in 50 % MeOH at concentrations of 2–10 mg/mL; CA was dissolved in 50 % ACN at concentrations of 2.5–10 mg/mL; and DPP-TFB was dissolved in 100 % MeOH at concentrations of 1–10 mg/mL. Variations in the solvent composition did not influence the efficiency of the on-tissue derivatization reaction. Trials were performed by spraying 1–5 layers using a flow rate of 10 μL/min with different derivatization reagents, using DHB as a matrix. The deposition of the derivatization reagent has a critical effect on the detection of amino metabolites, as shown in Supplementary Figure 2. The figure shows, as an example, the visualization of consecutive sagittal sections sprayed with increasing layers of TAHS (10 mg/mL) and CA (2 mg/mL). Initially an increase in the amount of derivatization reagent provides a higher intensity of derivatized amino metabolites (e.g. glyine-CA derivative). However, when excess derivatization reagent is added, the signal of the derivatization reagent itself can dominate, as shown for GABA-TAHS derivative. We, therefore, defined an optimum derivatization preparation by maximizing the detection sensitivity of the derivatized amino metabolites without the derivatization reagent as the mass spectrum’s base peak. Optimized concentration and spraying conditions for each derivatization reagent were: TAHS (5 mg/mL, 3 layers at 10 μL/min); CA (4 mg/mL, 3 layers at 10 μL/min); DPPTFB (5 mg/mL, 5 layers at 10 μL/min).

The influence of reaction time was assayed between 1 and 24 h for all derivatization reagents. For the highest detection sensitivity of the compounds, an incubation of approximately 22–24 h was found to be necessary for a complete derivatization reaction. Longer periods of time did not result in an increase in signal intensity.

The co-spraying of derivatization reagent and MALDI matrix has been reported to be, in some cases, an effective strategy to minimize loss of spatial resolution and increase the efficiency of the workflow (Manier et al. 2014). Matrices were simultaneously applied to the tissue sections in order to maintain consistent analyte extraction and crystallization conditions. To achieve the same amount of derivatization reagent and matrix as when they were sprayed separately, they were mixed together using the following concentrations: TAHS (2.1 mg/mL) and DHB (30 mg/mL) in 50 % ACN and 0.1 % TFA; CA (1.7 mg/mL) and DHB (30 mg/mL) in 50 % MeOH and 0.1 % TFA; and DPP-TFB (3.6 mg/mL) and DHB (30 mg/mL) in 80 % MeOH and 0.1 % TFA. The spraying method for DHB was used to deposit the combination of DHB and derivatization reagent and the slides were kept for 24 h at their corresponding temperature reactions. The resulting images did not reveal improvements in signal intensities or image quality. However, the derivatization of some amino compounds was much less effective. This could be explained by the fact that a DHB acidic solution is required for an effective ionization in positive mode, which gives an unsuitable derivatization reaction environment, which in most of cases, require soft basic pH conditions (Toue et al. 2014; Rebane et al. 2012; Shimbo et al. 2009).

In MALDI MSI the choice of matrix is critical for determining which molecules can be analysed and with which sensitivity. Some derivatization reagents, like TAHS and DPP-TFB, form positively charged derivatives that enable their analysis without the addition of matrix (Shariatgorji et al. 2014, 2015). To determine the best MSI method, consecutive tissue sections were analysed using DHB, CHCA, or in the absence of matrix. For DHB, the method described above was used, whereas for CHCA (5 mg/mL in 50 % ACN, 0.3 % TFA) was sprayed in 5 layers (1 layer at 5 μL/min, 1 layer at 15 μL/min, and 3 layers at 20 μL/min). The results showed a dramatic reduction in the signal of DPP-TFB derivatized amino metabolites in absence of matrix, severely limiting the detection of the derivatized metabolites. The MALDI matrices CHCA and DHB gave similar signal intensities for the derivatized amino metabolites. DHB was selected because the images displayed a slightly lower metabolite delocalization.

From these results we determined the optimal on-tissue derivatization procedures (cf. Sect. 2). In brief, brain tissue sections were sprayed using the three different derivatization reagents and left to react for 24 h at optimal temperatures. Subsequently, tissue sections were spray-coated with DHB and MALDI-MSI data acquired using ultra high mass resolution MALDI-FTICR-MSI.

### Visualization of derivatized amino metabolites in brain sections by MALDI-FTICR-MS

For the comparative study of the three different derivatization reagents, consecutive WT mouse brain sagittal sections were used. None of the amino metabolites could be visualized in the absence of derivatization reagent. The peaks of the derivatized compounds were not present in the matrix or tissue controls, which consisted of, respectively, a space adjacent to the tissue or a consecutive tissue section subjected to all the sample treatment but in absence of derivatization reagent. A representative spectra from the metabolites derivatized with the different derivatization reagents is shown in Supplementary Figure 3.

**Fig. 1:**
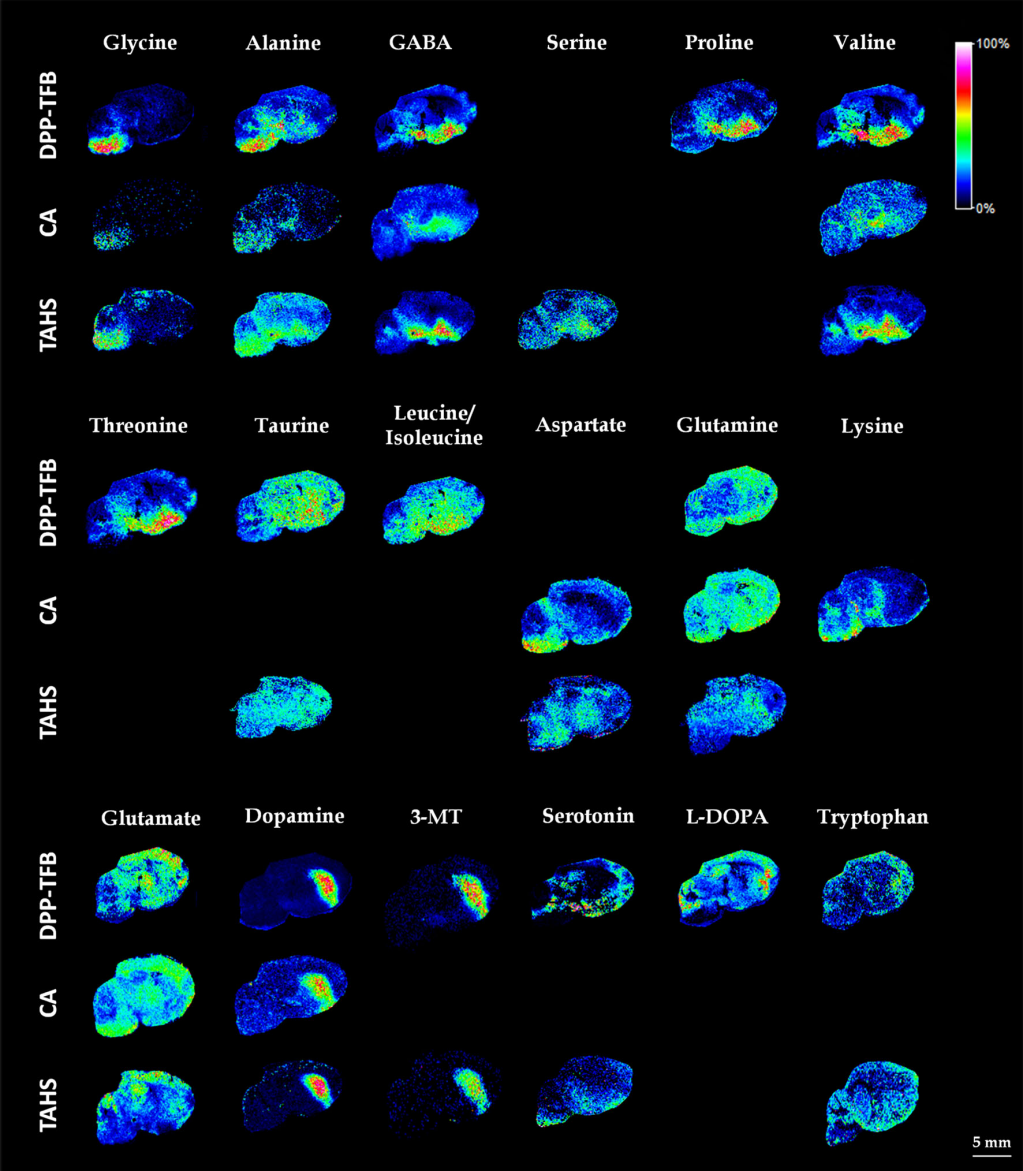
MSI visualizations of derivatives of amino metabolites with TAHS, CA, and DPP-TFB in mouse non-treated WT brain sagittal sections. The metabolite dataset was recorded in positive ion mode with a MALDI-FTICR and DHB as a matrix

The analysis was performed using ultra high resolution MALDI-FTICR-MS. Supplementary Table 1 shows the detected TAHS-, CA- and DPP-TFB-derivatized amino metabolites, including the theoretical and observed *m/z* values of each derivative. All mass errors were below 2 ppm using external calibration. Figure 1 shows a selection of images of TAHS-, CA- and DPP-TFB-derivatized amino metabolites in the mouse brain sections. A total of 18 compounds were detected. Some compounds were detected only using a specific derivatization reagent, while other compounds were detected with a range of reagents. Aliphatic amino acids such as alanine, glycine, leucine/isoleucine, proline, and valine were readily detected. In contrast, aromatic amino acids such as phenylalanine, tryptophan, and tyrosine were poorly or not detected. The detection of derivatives of basic amino acids (arginine, lysine, and histidine) has been reported to be extremely challenging (Toue et al. 2014). In line with this, arginine and histidine were not detected and lysine was detected only when using CA derivatization. In comparison, acidic amino acids (aspartic and glutamic acids) were readily detected, though this may also reflect their relatively higher concentration in brain tissue (Shariatgorji et al. 2014). For the DPP-TFB derivatives of the neurotransmitters aspartate and glutamate interference of matrix or derivatization reagent peaks was observed, leading to poorer quality images.

The visualization of the derivatized amino metabolites show, in several cases, high intensities and specific localizations. In several cases MALDI-FTICR-MS analysis verified the presence of signals consistent with the three different derivatives, like for glycine, alanine, GABA, valine, and dopamine. Distributions were also consistent with previously published data. For example GABA was found to be most abundant in hypothalamus, midbrain, and basal forebrain, which is consistent with previous reports (Alreja et al. 2000; Shariatgorji et al. 2014). GABA is a dominant NT in hypothalamus and plays an important role in hypothalamic inhibitory circuits (Decavel and Vandenpol 1990). Glutamate was detected in many structures of the brain but was mostly localized in striatal and cortical regions, in agreement with what has been published before (Shariatgorji et al. 2014).

**Fig. 2:**
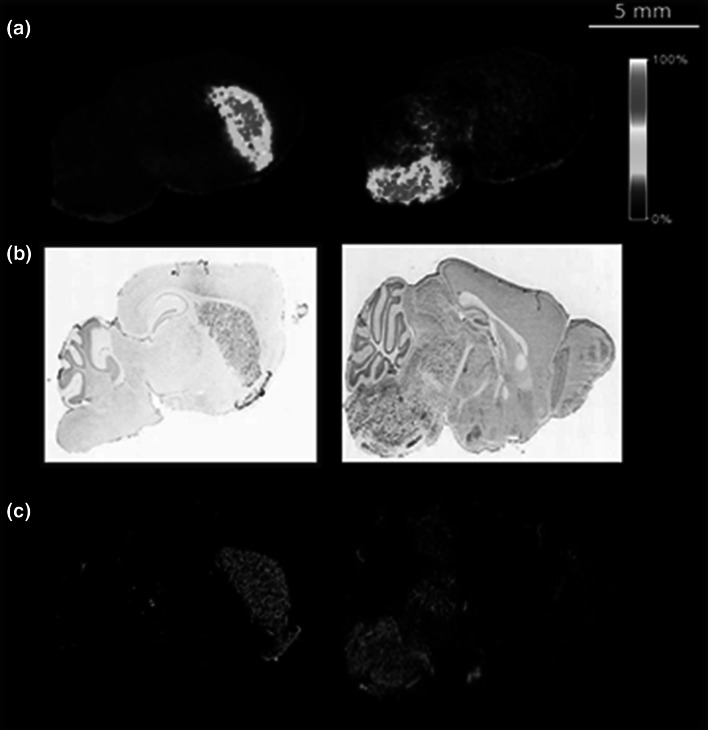
**a** MSI visualization for dopamine-DPPTFB derivative (*left*) and glycine-DPPTFB derivative (*right*) in non-treated WT mouse brain sagittal sections. **b** ISH visualization and; **c** FISH visualization of dopamine receptor D1A (*left*) and glycine receptor alpha 1 (*right*) from the Allen Brain Atlas (http://www.brain-map.org/)

Notably, the distribution observed for some derivatized amino metabolites is consistent with gene expression data contained in the Allen Brain Atlas (Lein et al. 2007) for their corresponding receptors. Clear examples of such correlation was provided for amino metabolites glycine and dopamine by comparing the MS images of DPP-TFB derivatives of metabolites dopamine and glycine to in situ hybridization (ISH) and fluorescent in situ hybridization (FISH) images of dopamine receptor D1A and glycine receptor alpha 1 subunit expression, respectively (Fig. 2). In the case of dopamine, both the dopamine derivative analysed by MALDI-FTICR-MS and its receptor show good agreement, being located mainly in the striatum, cortical subplate, and palladium. Notably, derivatized metabolite 3-methoxytyramine (3-MT), a metabolite of dopamine, is also localized in the same brain regions In case of glycine, the visualization of derivatives shows a localization in medulla and pons where also the glycine receptor, alpha 1 subunit is also expressed (Fig. 2).

This evaluation showed that for most amino compounds, the maximum MALDI signals and the best spatial distribution were obtained by spraying DPP-TFB dissolved in 100 % MeOH. This reaction produces charged N-alkyl pyridinium ions that are readily detected with high sensitivity in MALDI-MSI. However, in some specific cases like in the analysis of negatively charged ions other reagents showed better results. For that reason, we can conclude that the three derivatization reagents work in a somewhat complementary manner and that the use of a combination of different reagents can be a valuable strategy for the detection of a wider range of amino metabolites.

### Visualization of amino neurotransmitters changes in mouse brain that underwent CSD in one hemisphere

**Fig. 3:**
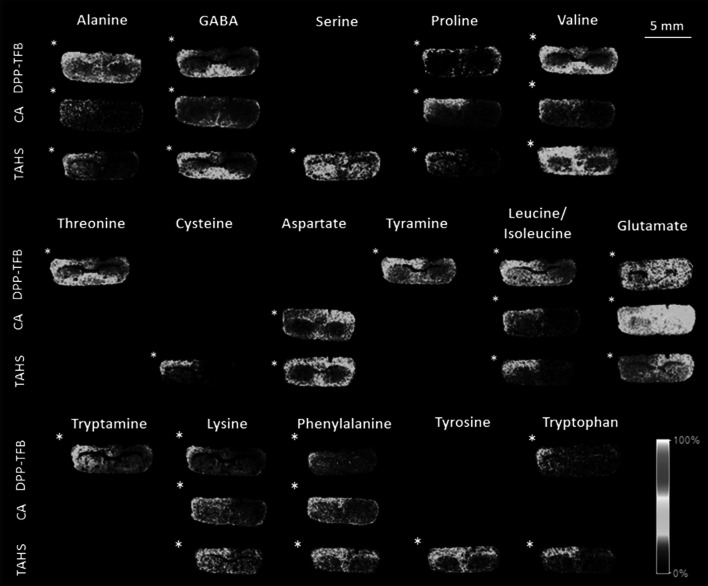
MSI datasets for TAHS-, CA-, and DPP-TFB-derivatives of amino metabolites after CSD in coronal brain sections from a wildtype mouse. *Asterisk* indicates the CSD hemisphere. The metabolite dataset was recorded as in Fig. 2

The combination of on-tissue derivatization strategies together with ultra high mass resolution MSI led us to investigate the chemical and spatial extent of disturbances in amino metabolites that follow CSD, evoked in one hemisphere leaving the other hemisphere as an internal control (Jones et al. 2012). Seven CSD events, spaced 5 min apart, were evoked in one hemisphere after which the animal was immediately sacrificed, the brain removed and heat stabilized using a Denator Stabilizor instrument. To differentiate changes related to the surgical procedure, but not to CSD, also an analysis of a SHAM-operated animal was performed. The SHAM animal underwent an identical surgical procedure except that KCl was replaced with NaCl, which does not induce a CSD (Shyti et al. 2015). 6 CSD and 6 SHAM brain sections were analysed, randomly sampled from the region between where CSD was evoked and recorded, to assess the reproducibility of the results. Furthermore, since the three derivatization methods gave complementary results and can be used to validate amino metabolite changes, they were all used for the analysis of mouse brain sections. Figure 3 shows the visualization of several derivatized amino metabolites obtained by MALDI-FTICR-MS in which the hemisphere in which CSD was induced is marked with an asterisk. The images reveal amino metabolites that showed clear contralateral changes (both increased and decreased signal intensities) after CSD, which were not observed in the SHAM experiments (Supplementary Figure 4). The increased signal of some amino metabolites after CSD allowed us to detect new compounds that were not detected under the SHAM condition, namely the metabolites cysteine, tyramine, tryptamine, phenylalanine, and tyrosine. Figure 3 shows clear differences between the CSD and the control hemisphere. Contralateral differences were evident in cortical structures, but less so in subcortical structures further away from the cortex, for all datasets, as observed previously (Jones et al. 2012). Several free amino metabolites (alanine, GABA, serine, proline, valine, cysteine, leucine/isoleucine, lysine, phenylalanine, tyrosine, tryptophan) were detected with increased intensities in the CSD hemisphere. On the other hand, aspartate and glutamate were found to be decreased in the CSD-affected hemisphere. No changes were observed for metabolites glycine, taurine, glutamine, dopamine, 3-MT, and L-DOPA (Supplementary Figure 5).

Disturbances in several amino metabolites have been previously associated with CSD both in brain tissue (Jones et al. 2012; Cavus et al. 2008; Levy and Degnan 2013; Fabricius et al. 1993) and body fluids (Ragginer et al. 2012; de Tommaso et al. 2012). Our MSI experiments revealed a significant increase in several amino metabolites in the cortex after CSD, notably GABA, an inhibitory neurotransmitter that counters the excitatory glutamate released by CSD. The intensities of the excitatory amino neurotransmitters glutamate and aspartate were found to be decreased in the CSD affected cortex. The release of excitatory neurotransmitters, particularly glutamate, is responsible for the initiation and propagation of CSD (Somjen 2001), and so the lower levels were at first glance contradictory but were consistently detected. However, it is known that glutamate is rapidly taken up by surrounding glial cells and converted to other metabolites such as glutamine and GABA (consistent with our data). Furthermore experimentally it has been found that evoking subsequent CSD events becomes more difficult. These observations all support the notion that the glutamate released by CSD is rapidly converted into alternative metabolites as a manner of countering the heightened cellular excitability.

## Concluding remarks

The combination of on-tissue chemical derivatization and accurate mass measurements using MALDI-FTICR-MS allowed us to achieve the necessary sensitivity and structural specificity for MSI of endogenous amino metabolites directly in mouse brain tissue. The combination of the three derivatization reagents enabled the recording of up to 23 amino metabolites distributions. Furthermore, the data demonstrate the ability of the technique to monitor monoamine neurotransmitters in the mouse brain under basal conditions, and after CSD that affects one hemisphere so the other hemisphere can act as an internal control. The determination of amino neurotransmitters dynamics is essential for understanding the pathophysiology of various neurological disorders such as migraine, stroke, depression, and epilepsy.

## Electronic supplementary material

Supplementary material 1 (DOCX 3400 kb)
