# Supplementary material for: Mass spectrometry imaging of amino neurotransmitters: a comparison of derivatization methods and application in mouse brain tissue
Source: Metabolomics. 2016 Jan 8;12:30. doi: 10.1007/s11306-015-0926-0 (PMC4705126; doi:10.1007/s11306-015-0926-0)
Supplement: Supplementary file 1 — Supplementary material 1 (DOCX 3400 kb) [file 11306_2015_926_MOESM1_ESM.docx]

**Supplementary Material**

**Supplementary Table 1**. Accurate mass measurement of derivatized amino metabolites on brain tissue sections using MALDI-FTICR-MS.

**Supplementary Figure 1.** Schematic derivatization reaction of an amino acid with **A**) TAHS, **B**) CA, and **C**) DPP-TFB.

**Supplementary Figure 2.** **A)** MSI visualization of *m/z* 236,1 corresponding to glycine-CA derivative using 1, 3, and 5 layers of CA (2 mg/mL, flow rate 10 µL/min), **B)** MSI visualization of *m/z* 280,2 corresponding to GABA-TAHS derivative using 1, 3, and 5 layers of TAHS (10 mg/mL, flow rate 10 µL/min).

**Supplementary Figure 3.** Representative spectra from different MSI datasets. **A)** In absence of derivatization reagent, **B)** TAHS-derivatives, **C)** CA-derivatives, **D)** DPP-TFB-derivatives.

**Supplementary Figure 4.** MSI datasets for TAHS-, CA-, and DPP-TFB-derivatives of amino metabolites in SHAM mouse cortical brain sections. ^*^ indicates the CSD hemisphere. The metabolite dataset was recorded as in **Figure 1**.

**Supplementary Figure 5.** MSI datasets for TAHS-, CA-, and DPP-TFB-derivatives of amino metabolites after CSD in wild type mouse cortical brain sections. ^*^ indicates the CSD hemisphere. The metabolite dataset was recorded as in **Figure 1**.

**Supplementary Table 1**. Accurate mass measurement of derivatized amino metabolites on brain tissue sections using MALDI-FTICR-MS.

| **Compound** | **THAS-derivative** | | | **CA-derivative** | | | **DPP-TFB-derivative** | | |
| --- | --- | --- | --- | --- | --- | --- | --- | --- | --- |
|  | **Theoretical** | **Observed** | **Error (ppm)** | **Theoretical** | **Observed** | **Error (ppm)** | **Theoretical** | **Observed** | **Error (ppm)** |
| **Glycine** | 252.1343 | 252.1341 | 0.79 | 236.0917 | 236.0916 | 0.42 | 290.1176 | 290.1173 | 1.03 |
| **Alanine** | 266.1499 | 266.1500 | -0.38 | 250.1074 | 250.1073 | 0.40 | 304.1332 | 304.1329 | 0.99 |
| **GABA** | 280.1656 | 280.1657 | -0.36 | 264.1230 | 264.1230 | 0.00 | 318.1489 | 318.1487 | 0.63 |
| **Serine** | 282.1448 | 282.1450 | -0.71 | 266.1023 | - | - | 320.1281 | 320.1277 | 1.25 |
| **Proline** | 292.1656 | 292.1655 | 0.34 | 276.1230 | 276.1230 | 0.00 | 330.1489 | 330.1487 | 0.61 |
| **Valine** | 294.1812 | 294.1811 | 0.34 | 278.1387 | 278.1385 | 0.65 | 332.1645 | 332.1646 | -0.30 |
| **Threonine** | 296.1605 | 296.1598 | 2.36 | 280.1180 | 280.1182 | -0.71 | 334.1438 | 334.1433 | 1.50 |
| **Cysteine** | 298.1220 | 298.1223 | -0.94 | 282.0795 | - | - | 336.1053 | - | - |
| **Taurine** | 302.1169 | 302.1167 | 0.83 | 286.0744 | 286.0743 | 0.35 | 340.1002 | 340.1003 | -0.29 |
| **Isoleucine/Leucine** | 308.1969 | 308.1970 | -0.29 | 292.1543 | 292.1542 | 0.34 | 346.1802 | 346.1799 | 0.87 |
| **Aspartate** | 310.1397 | 310.1399 | -0.64 | 294.0972 | 294.0972 | 0.00 | 348.1230 | - | - |
| **Tyramine** | 314.1863 | - | - | 298.1438 | - | - | 352.1696 | 352.1702 | -1.70 |
| **Glutamine** | 323.1714 | 323.1711 | 0.96 | 307.1288 | 307.1288 | 0.00 | 361.1547 | 361.1556 | -2.49 |
| **Lysine** | 323.2078 | 323.2081 | -0.93 | 307.1652 | 307.1656 | -1.30 | 361.1911 | 361.1909 | 0.55 |
| **Glutamate** | 324.1554 | 324.1553 | 0.46 | 308.1129 | 308.1126 | 0.97 | 362.1387 | 362.1383 | 1.10 |
| **Dopamine** | 330.1812 | 330.1809 | 0.94 | 314.1387 | 314.1384 | 0.95 | 368.1645 | 368.1645 | 0.00 |
| **Tryptamine** | 337.2023 | - | - | 321.1598 | - | - | 375.1856 | 375.1852 | 1.07 |
| **Phenylalanine** | 342.1812 | 342.1813 | -0.23 | 326.1387 | 326.1383 | 1.32 | 380.1645 | - | - |
| **3-MT** | 344.1969 | 344.1968 | 0.44 | 328.1543 | - | - | 382.1802 | 382.1801 | 0.26 |
| **Serotonin** | 353.1972 | - | - | 337.1547 | - | - | 391.1805 | 391.181 | -1.28 |
| **Tyrosine** | 358.1761 | 358.1764 | -0.84 | 342.1336 |  |  | 396.1594 | - | - |
| **L-DOPA** | 374.1710 | - | - | 358.1285 | - | - | 412.1543 | 412.1546 | -0.73 |
| **Tryptophan** | 381.1921 | 381.1915 | 1.57 | 365.1496 | - | - | 419.1754 | 419.1761 | -1.67 |

**Supplementary Figure 1**


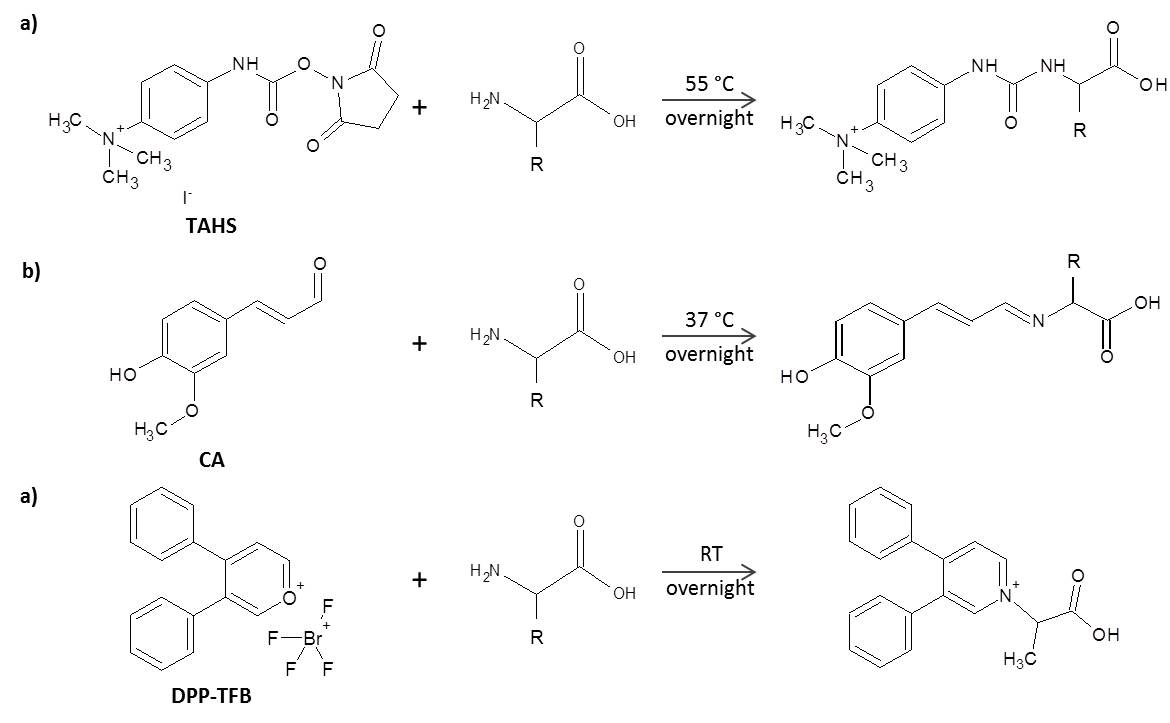


**Supplementary Figure 2**

**Supplementary Figure 3**

**Supplementary Figure 4**

**Supplementary Figure 5**
